# The interplay among sleep patterns, social habits, and environmental cues: insights from the Spanish population and implications for aligning daily rhythms

**DOI:** 10.3389/fphys.2024.1323127

**Published:** 2024-10-25

**Authors:** María-Ángeles Bonmatí-Carrión, Jesús Vicente-Martínez, Juan Antonio Madrid, Maria Angeles Rol

**Affiliations:** ^1^ Chronobiology and Sleep Laboratory, Department of Physiology, College of Biology, University of Murcia, Mare Nostrum Campus, IUIE, IMIB-Arrixaca, Murcia, Spain; ^2^ Ciber Fragilidad y Envejecimiento Saludable (CIBERFES), Instituto de Salud Carlos III, Madrid, Spain

**Keywords:** sleep, time use, circadian synchronization, desynchronization, three times, environmental time, social time, sleep-related habits

## Abstract

**Introduction:**

The interplay among sleep patterns, social habits and environmental cues is becoming increasingly more important for public health and wellbeing due to its connection to circadian desynchronization. This paper explores said connections in Spain (which has an official and solar time mismatch), introducing the “Three Times Score” ‒which is based on questions widely used in the field‒ as a complementary tool for exploring the interplay of daily rhythms.

**Methods:**

The questionnaire covers sleep-related habits, social time, and environmental time. The study includes 9,947 participants (34.89 ± 12.15 y/o, mean ± SD; 5,561 women) from different Spanish regions. Sleep parameters were obtained for work and free days, as well as a modified version of the sleep-corrected midsleep on free days (MBFbc) and a parameter similar to social jet lag, both derived from bed time rather than sleep time. A number of indexes were computed to compare bed and work-related habits, together with natural light/dark cycle, along with the Three Times Score. Mixed-effect regression analysis was used to test whether the biological, social and environmental factors included in the study significantly predicted the sleep-related parameters: bedtime, wake-up time, time in bed and mid-bedtime.

**Results and discussion:**

Temporal differences were found between work and free days, with waking-up occurring 2 h earlier on work days (7:10 ± 0:01) than on free days (9:15 ± 0:01). Bed times were 1 h earlier on work days (23:46 ± 0:01) than on free days (00:45 ± 0:01), whereas time in bed was over 1 h shorter on work (7 h 23 min) *versus* free (8 h 29 min) days. Strong correlations were found between work starting time and waking-up and bedtimes on workdays. Women went to bed earlier and woke up later, spending more time in bed. Differences in sleep habits were observed between work and free days across all age groups. The group of younger adults (18–30) reported going to bed later than older and younger groups, especially on free days. Adolescents and young adults also woke-up later than other age groups, especially on free days. Social jet lag (relative to bed time) and desynchronization indexes also varied with age, with younger adult participants exhibiting higher levels. Seasonal differences were limited, with minor variations between winter and summer. According to the multiple regression analysis, social (day type, work start time, alarm clock usage), biological (age, gender, in most cases related to sex) and environmental (sunset time) factors significantly contribute to predicting sleep/bed related schedules. This study provides insights into sleep habits in the Spanish population, introducing the Three Times Score as a complementary tool for exploring the interactions between sleep/bed-related habits, natural darkness and work-related schedules. Understanding this interplay is crucial for developing tailored interventions to improve sleep and wellbeing.

## 1 Introduction

Human circadian rhythmicity is influenced by three time frames: internal, environmental and social times. Each of them plays a crucial role in regulating our daily lives, impacting the temporal organization of all physiological processes (Roenneberg et al., 2019b).

Internal time, also referred to as endogenous time, encompasses the intrinsic biological rhythms that are generated within our bodies. The master pacemaker of internal time is the suprachiasmatic nucleus (SCN), a small region in the hypothalamus that synchronizes other peripheral clocks located in all organs and tissues. This system regulates a multitude of physiological processes and behaviors. However, since the endogenous period deviates from 24 h, this organized structure of oscillators must be reset daily, mainly through the effect of the light-dark cycle on the central pacemaker. Additional inputs include schedules for physical activity and meals, which hold particular relevance for peripheral oscillators. This system also produces measurable outputs, such as the sleep-wake cycle, hormonal secretions, motor activity and body posture. Notably, some of these outputs can also serve as inputs to the system, as is the case for the sleep-wake cycle (Bonmati-Carrion and Tomas-Loba, 2021).

Closely related to the internal time is the concept of chronotype, which refers to the individual differences in the timing of biological rhythms, particularly the sleep-wake cycle. It determines whether a person is more prone to be a “morning person” (early chronotype) or an “evening person” (late chronotype). Chronotype is known to be influenced by biological (genetics, age and sex) (Roenneberg et al., 2007; Fischer et al., 2017), social (official country time) and environmental factors (light-dark cycle) (Roenneberg et al., 2007).

Indeed, environmental time refers to the natural cycles that are inherent in our surrounding environment. One of the most prominent environmental factors that influences human rhythmicity is the alternation between day and night, driven by the Earth’s rotation, which interacts with the internal time by synchronizing and entraining it. Thus, regular exposure to bright light in the morning can advance the timing of our sleep-wake cycle, making individuals more inclined towards early chronotypes. Conversely, exposure to light in the evening can delay the circadian rhythm, leading to a propensity for late chronotypes (Zeitzer et al., 2000; Khalsa et al., 2003; Bonmati-Carrion et al., 2014; Papatsimpa et al., 2021).

The interaction between internal and environmental time is affected to a large extent by social time, which refers to the societal and cultural norms that dictate the organization of daily activities and routines. It includes the concept of official time, which is standardized and used for practical purposes, like scheduling events and coordinating activities within a community. Interestingly, even though official time is originally based on geographical location, certain regions may deviate from their expected time zone due to socio-economic or historical reasons. For example, Spain follows Central European Time (CET, GMT+1 during standard time, GMT+2 during daylight saving time), even though, based on its geographical location, it should align with Western European Time (WET, GMT0). Official time, together with social routines, very much influence the light-dark exposure (Bonmatí-Carrión et al., 2022). Humans normally have daily routines related to duties, such as school or work, that are synchronized to the official time, so both factors constitute a third timeframe influencing circadian rhythmicity.

Internal, environmental and social times interact on a daily basis, influencing each other and potentiating the circadian alignment or, on the contrary, the circadian desynchronization leading to a misalignment of circadian rhythms (Roenneberg et al., 2019b) with detrimental effects on health and wellbeing. This interaction has been demonstrated in epidemiological studies and natural experiments considering geographical longitude and time zones (Borisenkov, 2011; Gu et al., 2017; Caporaso et al., 2018; Bonmatí-Carrión et al., 2022).

The interplay between sleep patterns on both work and free days has relevant implications for human health and wellbeing. Work schedules and social obligations often lead to discrepancies in sleep timing (Bonmatí-Carrión et al., 2022), resulting in what is known as “social jetlag”. Social jetlag refers to the misalignment between an individual’s biological clock and their social or work-related schedule (Wittmann et al., 2006). This misalignment can result in disrupted sleep patterns, reduced sleep quality, and increased sleep deprivation. Moreover, social jet lag has been associated with various health issues, such as cardiovascular diseases, metabolic disorders (McMahon et al., 2019), impaired cognitive function (Taillard et al., 2021) and psychiatric conditions (Levandovski et al., 2011). Understanding how our chronotype, social duties and exposure to light interact can help to optimize our daily routines, sleep quality, and overall wellbeing. Despite its potential impact on public health, there remains a research gap in understanding the prevalence and consequences of social jetlag within specific populations, especially when a gap between geographical location and official time occurs, as in the case of Spain.

Thus, the aim of this article was to explore the sleep-related and social habits in relation to work schedules in a population residing in Spain. An additional goal of the study was to assess sleep-related habits while considering work and free days separately, in order to analyze the impact of social time in the particular context of Spain, where the official time is misaligned with its corresponding geographical time zone. We also propose different indexes to objectively assess the interplay among sleep/bed-related habits, natural darkness and work-related time frames.

## 2 Materials and methods

### 2.1 Questionnaire and recruitment

The “Three-Times Questionnaire”, based on the Munich Chronotype Questionnaire [MCTQ, (Roenneberg et al., 2003)], has been available on the Chronobiology Laboratory website (Spanish version: https://www.um.es/cronobiologia/taller-del-relojero/autoevaluacion/test-tres-tiempos/; English version: https://www.um.es/cronobiologia/en/watchmakers-studio/self-assessment/three-times-test/) since October 2016. The survey link was made publicly accessible through our communication channels, including the official website and social media platforms. The Spanish Society of Sleep also disseminated the questionnaire through its own channels, including press releases, encouraging participation from individuals of different backgrounds and interests. Although no power or sample size calculation was performed *a priori*, the data collection for this study was closed in 2023, once the raw dataset reached >20,000 responses.

The questionnaire used required the following information to be introduced manually: age (years old), gender (woman/man), height (in cm), weight (in kg), country and city. The system automatically recorded the IP address, date and time of data collection. The questionnaire was divided into three sections, which asked for information about sleep-related habits; social time (work-related habits); and environmental time (natural light/dark cycle information).

#### 2.1.1 Section 1. sleep/bed-related habits

In this section, questions about bed and sleep-related habits were included for both work days and free days, specifically bedtime and wake-up time. Although the time of sleep onset could also provide valuable information, based on our experience with volunteers, bedtimes and wake-up times are easier to recall and self-report than sleep onset or sleep latency, which makes it easier for participants to complete the entire questionnaire.

Work days
I go to bed at ____.
I wake up at ____ (with/without an alarm).
Free days
I go to bed at ____.
I wake up at ____ (with/without alarm).

#### 2.1.2 Section 2. social time

I start working at ____.
I finish working at ____.
It takes me ____ hours to get to work (from home).
I need ____ hours to get ready to leave home.
I work ____ days a week.

#### 2.1.3 Section 3. environmental time

Although the system asked participants to include sunrise and sunset times, these data were subsequently revised and calculated by the authors in order to assure precise data. Geographical coordinates were obtained from the town indicated in the questionnaire, together with the date of the response, and sunrise and sunset were subsequently calculated from these data.

### 2.2 Filtering

We obtained an original dataset containing 24,080 rows. In order to limit the data to that from participants residing in Spain and eliminate incomplete or erroneous entries, we applied different filters to the original dataset, in order to exclude those rows that included one or more of the following situations:

• Duplicate IP address [5015].
• Responses received from countries other than Spain [5669].
• Incorrect spelling of the city that hampered automatic association with the corresponding geographical coordinates (needed for sunrise and sunset times) [1564].
• Missing response for one or more items related to bed/wake-up times [77].
• Weight <30 kg or height >210 cm or height <120 cm, age >116 and age <13 [124].
• Time in bed longer than 12 h or shorter than 3 h (considering the bedtime and wake-up times included in the questionnaire) [50].
• The sum of the time spent to get to work from home, and the time needed to get ready to leave home was longer than the difference between work start time and wake-up time [1255].
• Work started later than 20:00 and work ended earlier than 08:00, in order to exclude potential night shift workers [377].

### 2.3 Study population

**FIGURE 1:**
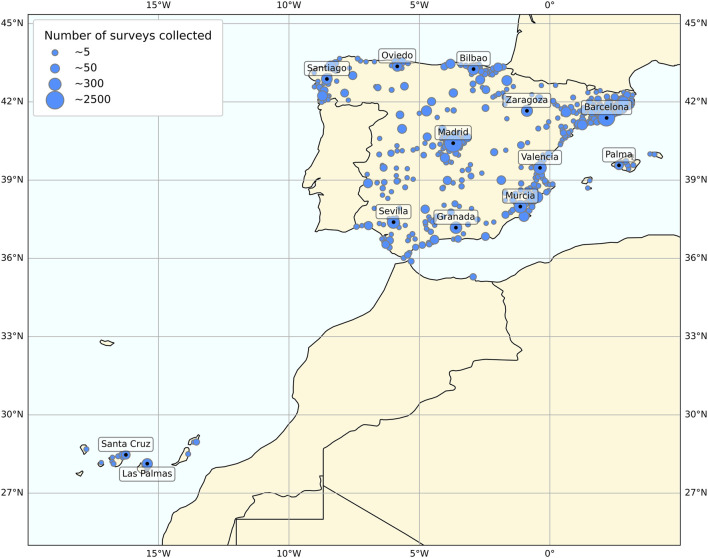
Map of Spain representing the geographical locations of the participants. Circle size represents the number of questionnaires answered from each area.

After completing the filtering process, 9,947 participants residing in Spain (34.89 ± 12.15 y/o, mean ± SD; 5,561 women) were included. This research project was approved by the University of Murcia Ethics Committee (ID 2072/2018), and all research was performed in accordance with relevant guidelines/regulations. As Figure 1 shows, the geographical location of the participants covered a reasonable proportion of Spain, including the Balearic and Canary Islands.

For the study of the age effect, the population was divided into six groups: 13–17 y/o (adolescents), 18–30 y/o, 31–40 y/o, 41–50 y/o, 51–64 y/o and 65–80 y/o (probably retired from remunerated obligations).

### 2.4 Bed-related and time parameters, desynchronizations and the three times score

From the data collected, we calculated the following parameters both for work and free days:

Bed Time=I go to bed at…

Wake‐Up Time=I wake up at…

Time in Bed=Wake‐Up Time−Bed Time

$$Mid\;Bed\;Time=Bed\;Time+\frac{\left(Wake‐Up\;Time-Bed\;Time\right)}{2}$$

In addition, the average Time in Bed was calculated as the weighted average of time in bed on work and free days, considering the number of both types of days each participant reported having in a week.

$$Averaged\;Time\;in\;Bed=\left[\left(\left(\;Time\;in\;Bed\;\left(work\;days\right)*Number\;of\;work\;days\right)\;+\left(\;Time\;in\;Bed\;\left(free\;days\right)*Number\;of\;free\;days\right)\right)\right]/7$$

MBF_bc_ (Mid-time in Bed on Free days, with correction for work days), a modified version (and not equivalent) of midsleep on free days, corrected for sleep debt on work days (MSF_sc_) was also calculated as previously proposed by (Roenneberg et al., 2019a). Here, we replaced sleep onset with bed time and sleep duration by time in bed, as indicated below:

If Time in Bed (free days) ≤ Time in Bed (work days):

MBFbc=Mid Bed Time

If Time in Bed (free days) > Time in Bed (work days):

$$MBFbc=Bed\;Time\;\left(free\;days\right)+\frac{Average\;Time\;in\;Bed}{2}$$

#### 2.4.1 Desynchronizations and three times score calculations

For the calculation of the three desynchronization indexes (Bed-Work Desynchronization, BWD; Bed-Natural Darkness Desynchronization, BDD; and Work-Natural Darkness Desynchronization, WDD) and the three times score (a composed variable of those desynchronization indexes), we first calculated the following parameters related to bed time, social time and environmental time:

##### 2.4.1.1 Bed time related calculations

Bed-Central Time (bCT) is a measure of the midpoint of the time elapsed between bed time and wake up time, adjusted for differences between free days and workdays.

Similar to the correction made in Munich Chronotype Questionnaire (Roenneberg et al., 2019a), we also implemented the following correction:

When Time in Bed in free days < Time in Bed in work days:

Bed-Central Time - > Mid Bed Time (free days)

When Time in Bed in free days > Time in Bed in work days:

$$\text{Bed}-\text{Central}\;\text{Time}=Mid\;Bed\;Time\;\left(free\;days\right)-\frac{Time\;in\;Bed\;\left(free\;days\right)-Time\;in\;Bed\;\left(work\;days\right)}{2}$$

Example:
Age: 28 years/old.
Gender: Man.
City: Cartagena (Spain)
Wake-up time in work days: 7:30 (7.5 h in decimal format)
Wake-up time in free days: 9:00 (9.0 h in decimal format)
Bed time in free days: 00:00 (0/24 h in decimal format)
Time in Bed in work days: 8.5 h.
Time in Bed in free days: 9 h.
Mid Bed Time (free days) = (0 + 9)/2 = 4.5 (04:30)
bCT = 4.5 – (9–8.5)/2 = 4.25 (04:15)

##### 2.4.1.2 Social time related calculations

Initial Social Time (iST) and Final Social Time (fST) reflect the times associated with work-related schedules, including work start and end, as well as commute and preparation times.

Initial Social Time (iST)

It makes reference to the theoretical latest bCT that is compatible with work start time.

$$iST=Work\;Start\;Time-Commute\;Time\;\left(h\right)-Preparation\;Time\;\left(h\right)-\frac{Recommended\;Sleep\;Duration}{2}$$

Recommended Sleep Duration was based on previous recommendations issued by the National Sleep Foundation (Hirshkowitz et al., 2015) and the American Academy of Sleep Medicine (Watson et al., 2015) and were applied considering each individual age: <1 year old: 14.5 h; 1–3 years old: 13 h; 3–5 years old: 12 h; 5–12 years old: 10.5 h; 12–18 years old: 8.8 h; >18 years old: 8 h).

Example:
Work start time: 09:30 (9.5 h in decimal format)
Work end time: 17:00 (17 h in decimal format)
Commute time: 45 min (0.75 h)
Preparation time: 1 h
iST = 9.5 – 0.75 – 1 – (8/2) = 3.75.
Final Social Time (fST) and corrected Final Social Time (cfST):

It is calculated as the theoretical earliest bCT that is compatible with the end of the workday. For its calculation, commute time and a minimum disconnection and sleep preparation time of 1 h are taken into account.

$$fST=Work\;End\;Time+Commute\;Time\;\left(h\right)+1+\frac{Recommended\;Sleep\;Duration}{2}$$

If *f*ST > 24, cfST=fST−24

If *f*ST< 24, cfST=fST

Example:
fST = 17 + 0.75 + 1 + (8/2) = 22.75 = cfST.
Social Time Range (STR) and corrected Social Time Range (cSTR)

Social Time Range measures the lapse between iST and fST. Considering the circular nature of time, STR is corrected (cSTR) by adding 24 h when result is negative. It is the range of hours during which the bCT could occur so that the recommended sleep duration is compatible with the work start and end times.

STR=iST−fST

If STR < 0, $cSTR=\left(iST+24\right)-fST$

If STR > 0, cSTR=STR

Example:
STR = 3.75 – 22.75 = −19 (<0)
cSTR = (3.75 + 24) – 22.75 = 5

Mid-point of Social Time (MidST) represents the midpoint of adjusted social time, calculated from the corrected final Social Time (fST) and the corrected Social Time Range (cSTR).

$$MidST=cfST+\frac{cSTR}{2}$$

If MidST < 24, cMidST

If MidST > 24, cMidST=MidST−24

Example:
MidST = 22.75 + (5/2) = 25.25
cMidST = 25.25 – 24 = 1.25.

##### 2.4.1.3 Environmental time related calculations:

Environmental Time (ET) measures the central time based on natural darkness, calculated from sunrise and sunset times according to geographical coordinates.

$$ET=\frac{Sunrise\;Time+24-Sunset\;Time}{2}+Sunset\;Time$$

If ET > 24, cET=ET−24

If ET < 24, cET=ET

Example:
Sunrise time: 8:25 (8.42 h in decimal format)
Sunset time: 19:11 (19.18 h in decimal format)
ET = (8.42 + 24 – 19.18)/2 + 19.18 = 25.80
cET = 25.8 – 24 = 1.80.

##### 2.4.1.4 Desynchronizations

These measures evaluate the mismatch between bedtime, social time, and natural darkness:

• Bed-Work Desynchronization (BWD) measures the absolute difference between the midpoint of social time range (MidST) and the midpoint of the lapse between bed time and wake up time (bCT). It reflects the discrepancy between bedtime and work hours.

$$BWD=\left|MidST-bCT\right|\;hours$$

If bCT does not fall outside the window constrained by the Social Time Range (considering both work start and end restrictions), the BWD will be zero. However, if b-CT falls outside the Social Time Range constrained by the work schedule (start/end), BWD will be taken into account. This desynchronization will be calculated as the number of hours that b-CT falls outside the time window available due to work restrictions, considering only workdays during the week.

If BWD > $\frac{cSTR}{2}$ ,

$cBWD=\left(BWD-\frac{cSTR\;}{2}\right)x\;number\;of\;work\;days\;a\;week/7$

If BWD < $\frac{cSTR}{2}$ , cBWD=0

Example: BWD =|1.25 – 4.25 | = 3 h (> $\left.\frac{cSTR}{2}\right)$

cBWD = $\left(3-\frac{5\;}{2}\right)x\frac{5}{7}=0.36$

• Bed-Natural Darkness Desynchronization (BDD) is calculated as the absolute difference between the midpoint of the lapse between bed time and wake up time (bCT) and the midpoint of natural darkness (calculated as corrected environmental time, cET. It indicates the mismatch between bedtime and the natural darkness period.

$$BDD=\left|bCT-cET\right|\;hours$$

Example: BDD = |4.25 – 1.80 | = 2.45 h.

• Work-Natural Darkness Desynchronization (WDD) is calculated as the absolute difference between the midpoint of social time range (MidST) and the midpoint of natural darkness period (cET). It measures the mismatch between work-related schedule and the natural darkness cycle.

$$WDD=\left|MidST-cET\right|\;hours$$

Similar to BWD, if cET does not fall outside the window constrained by the Social Time Range (considering both the work start and end restrictions), WDD will be zero. However, if cET falls outside the Social Time Range constrained by the work schedule (start and/or end), WDD will occur. This desynchronization will be calculated as the number of hours that cET falls outside the time window available due to the work restriction, considering only the workdays during the week:

If WDD > $\frac{cSTR}{2}$ ,

$cWDD=\left(WDD-\frac{cSTR\;}{2}\right)x\;number\;of\;work\;days\;a\;week/7$

If WDD < $\frac{cSTR}{2}$ , cWDD=0

Example: WDD =|1.25 – 1.80 | = 0.55 h (< $\frac{cSTR}{2}$ ) - > cWDD = 0

• The Three Times Score combines the desynchronizations to provide an overall score indicating the degree of misalignment in bed time relative to work schedule and natural darkness. Since according to the circular nature of time, the maximal distance among two time points is 12 h either clockwise direction or counter clockwise, the maximal theorical misalignment for Bed-Work Desynchronization, Bed-Natural Darkness Desynchronization and Work-Natural Darkness Desynchronization is 12 h (thus 36 h jointly, considering normalization).

$$3T\;Score=\frac{BWD+BDD+WDD\;}{36}$$

Example:

$$3T\;Score=\frac{0.36+2.45+0\;}{36}=0.08$$

### 2.5 Statistical analyses

The time variables have been treated as circular in all calculations. The normality of the data was checked using a Kolmogorov-Smirnov test. Although visual inspection of the histograms revealed a distribution close to normal in most cases, most parameters were not confirmed as normal according to this test, so a non-parametric Mann-Whitney U test was used to compare men vs. women, winter vs. summer or daylight-saving time vs. standard time, while a Wilcoxon test was used to compare work days vs. free days within each class of variables. In comparisons by gender, we implemented age as a covariate to avoid a possible bias between the groups due to that factor. For comparisons among more than two groups (e.g., age groups), the Kruskal-Wallis test was performed. Spearman’s correlations were also performed on data directly reported by participants (bed/wake-up times). The significance level at *p* < 0.05 was Bonferroni-corrected for each variable, and all results in tables were expressed as the mean ± standard error of the mean (SEM). Additionally, the effect size was calculated based on the Wilcoxon signed-rank test, using the formula: r = Z/√n. Effect sizes below 0.3 were considered small, 0.3–0.5 were considered moderate, and values greater than 0.5 were considered large. A mixed-effect regression analysis (with participant identification as random effect) was conducted to predict bedtime, wake-up time, time spent in bed and mid-bed timing. The analysis included biological factors (age and sex inferred from gender, the latter treated as a dichotomous variable), social factors (work start time, work end time, and day type treated as dichotomous variables), and environmental factors (sunrise and sunset times, and official time treated as dichotomous variables, DST or ST). Likelihood ratio was used to assess the significance of the explanatory variables and sensitivity analysis post-hoc were performed according to the Akaike information criterion (AIC). All calculations and statistical analyses were performed using SAS version 9.4 and R software. Time data on figures is expressed in decimal format, while in the manuscript text they are expressed as hh:mm to facilitate text comprehension.

## 3 Results

Regarding the sleep-related habits of this population, we observed temporal differences between work and free days, especially in reported wake-up times. On work days, participants woke up 2 h earlier (07:10 ± 00:01) as compared to free days (09:15 ± 00:01). Although the difference in bed times between day types was smaller than that for the reported wake-up times (less than 1 h), it still occurred earlier on work days (23:46 ± 0:01) as compared to free days (00:45 ± 0:01) (*p* < 0.0001). Considering the reported bed time and wake-up times, individuals in this population spent on average more than 1 h longer in bed on free days (8 h 29 min) than on workdays (7 h 23 min) (*p* < 0.0001). When calculating a weighted week average taking into account the number of workdays, the time spent in bed for this population was 7 h 41 min. These reported sleep-related patterns also yielded differences in mid-bedtime (a proxy for midpoint of sleep) between work days (3:28 ± 0:00) and free days (5:00 ± 0:00) (*p* < 0.0001).

### 3.1 Gender dimension

**FIGURE 2:**
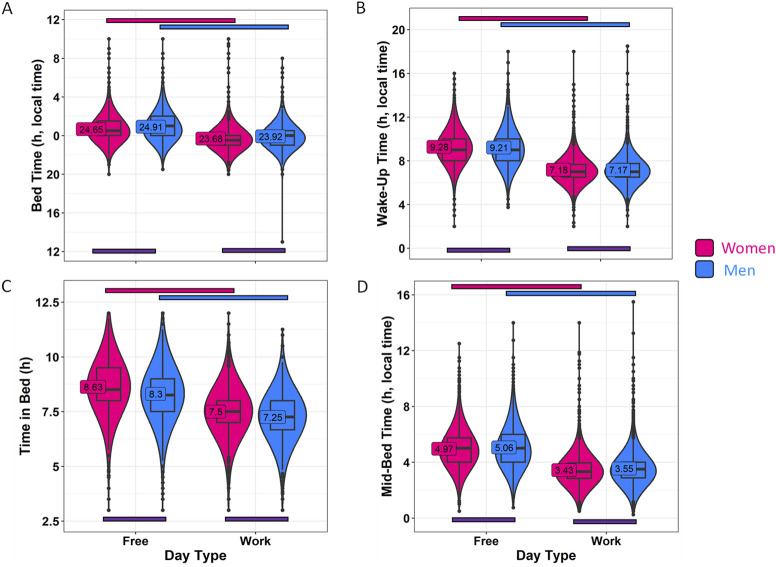
Sleep habits on workdays and free days, divided by gender. **(A)** Bed time, **(B)** wake-up time, **(C)** time in bed and **(D)** mid-bedtime in women (pink) and men (blue). Violin plots represent the kernel density estimation, with median, first and third quartiles represented in box plots. The mean is indicated as a number in decimal format. The Mann-Whitney U test was used to compare men vs. women, and significant differences between genders within the same day type are indicated by purple horizontal bars (*p* < 0.006). A Wilcoxon test was used to compare work vs. free days within each gender. Pink and blue horizontal bars indicate statistically significant differences between free and work days in women and men, respectively (*p* < 0.0001).

**FIGURE 3:**
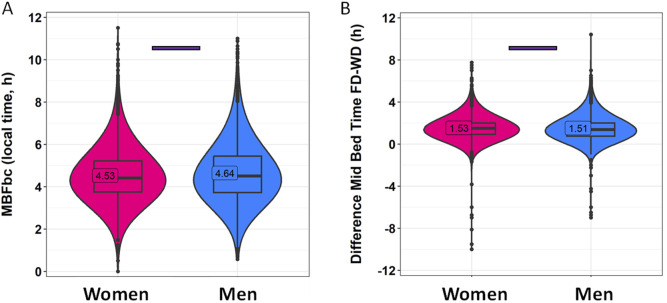
MBFbc **(A)** and difference in mid-bedtime between free and work days **(B)**, divided by gender. Violin plots represent the kernel density estimation, with median, first and third quartiles represented in box plots. The mean is indicated as a number in decimal format. The Mann-Whitney U test was used to compare men vs. women. The Kruskal-Wallis test was performed to compare age groups; significant differences among them are indicated by color-coded horizontal bars. Significant differences between genders are indicated by horizontal bars (*p* < 0.0001).

Sleep-related habits were also analyzed from a gender perspective (Figure 2; Supplementary Table S1). Female and male participants were of significantly different ages, with women being 33.52 ± 12.11 years old, while men were 36.63 ± 11.98 years old (mean ± SD). Although the range is similar, the statistical analyses in this section include age as a covariate. In this context, men reported going to bed 15 min later than women on both work and free days (Figure 2A; Supplementary Table S1) (*p* < 0.001). The differences in wake-up time (Figure 2B) were smaller, with men reporting waking up only 4 min earlier than women on free days (*p* < 0.0001). Considering these reported patterns, women also spent more time in bed than men on both free days (19 min, on average) and work days (15 min, on average) (*p* < 0.0001) (Figure 2C). The timing marker mid-bedtime (Figure 2D) occurred 5 and 7 min earlier in women as compared to men on free (*p* < 0.0001) and work days (*p* < 0.0001). MBFbc, a calculated parameter similar to MSFsc (Figure 3A; Supplementary Table S2), also exhibited these gender differences, with women showing slightly advanced times (04:31 ± 00:01) as compared to men (04:39 ± 00:01) (*p* < 0.0001). When comparing the weighted average for time in bed (Supplementary Table S2), women spent 7 h 48 min in bed, while men spent an average of 7 h 32 min in bed (*p* < 0.0001). We also calculated the variation in mid-bedtimes between free and work days (similar to social jet lag), obtaining a difference of 1.53 ± 0.01 h for women and 1.51 ± 0.02 h for men (*p* = 0.0008) (Figure 3B; Supplementary Table S2). In both women and men, the effect size of day type was moderate for bed time (0.365 for men; 0.396 for women) and time in bed (0.463 for men; 0.491 for women), while it was large (>0.5) for wake-up time (0.613 for men; 0.667 for women) and mid-bedtime (0.548 for men; 0.607 for women).

**FIGURE 4:**
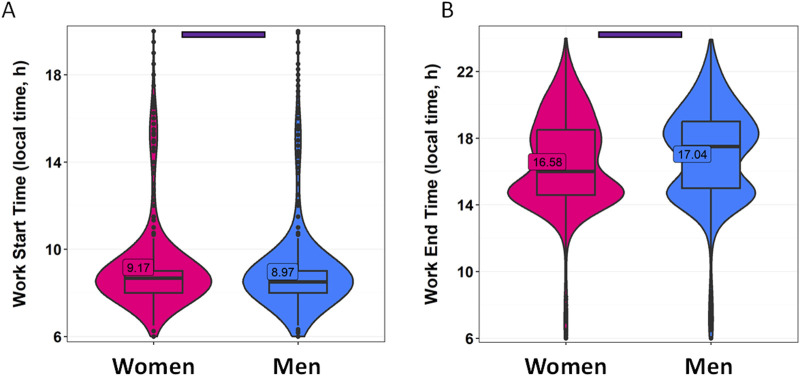
Work schedules divided by gender. **(A)** Work start time and **(B)** work end time in women (pink) and men (blue). Violin plots represent the kernel density estimation, with median, first and third quartiles represented in box plots. The mean is indicated as a number in decimal format. The Mann-Whitney U test was used to compare men vs. women; significant differences between genders are indicated by purple horizontal bars (*p* < 0.0001).

Work schedules also play a significant role in terms of human rhythmicity (Figure 4). In this case, we also divide the data by gender. Women included in this study reported starting work at 9:10 while men began on average at 8:58 (*p* = 0.0004) (Figure 4A; Supplementary Table S2). The average work end time was 16:34 for women and 17:02 for men (*p* < 0.0001) (Figure 4B; Supplementary Table S2). Interestingly, it is worth noting that the distribution of work end times is clearly inverted in women with respect to men, with a higher frequency of men finishing work later than women. Strong correlations were found between reported work start time and wake-up time (R = 0.670, *p* < 0.0001) and bed time (R = 0.425, *p* < 0.0001) on work days. The correlations of work start time with wake-up time (R = 0.249, *p* < 0.0001) and bed time (R = 0.271, *p* < 0.0001) were weaker on free days (Supplementary Figure S1).

The questionnaire we used also included questions about the time spent getting ready and the commute duration from home to the workplace (Supplementary Table S2). In this case, we observed that women spent approximately 9 min longer getting ready (56.54 ± 0.46 min) as compared to men (47.95 ± 0.50 min) (*p* < 0.0001). The time spent commuting was similar, with only 2 min of difference between the two genders (*p* < 0.0001); women averaged 29.00 ± 0.29 min, while men averaged 26.52 ± 0.30 min.

**FIGURE 5:**
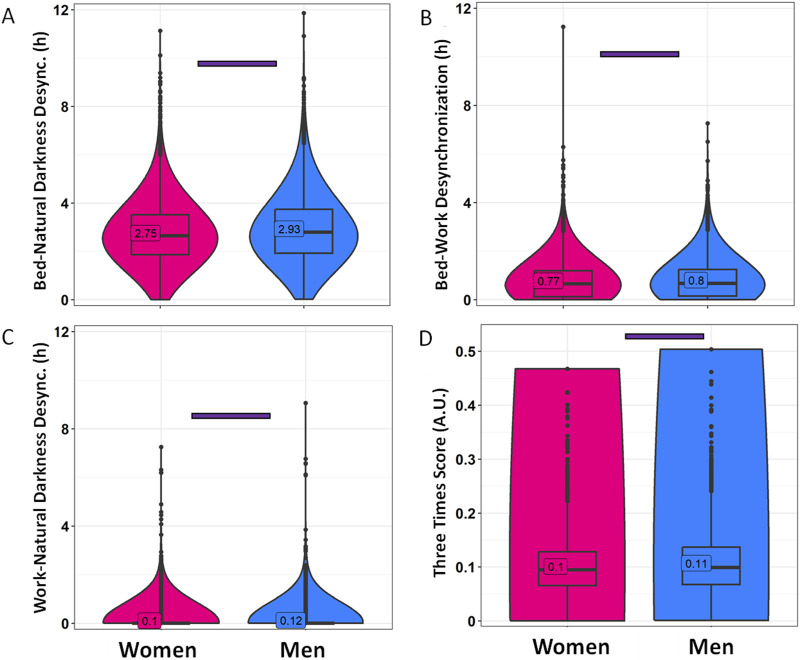
Desynchronization indexes divided by gender. Bed-natural darkness **(A)**, bed-work **(B)**, work-natural darkness **(C)** desynchronizations and Three Times Score **(D)** in women (pink) and men (blue). Violin plots represent the kernel density estimation, with median, first and third quartiles represented in box plots. The mean is indicated as a number in decimal format. The Mann-Whitney U test was used to compare men vs. women; significant differences between genders are indicated by purple horizontal bars (*p* < 0.0003).

Regarding the desynchronization indexes calculated (Figure 5; Supplementary Table S2), only the bed-natural darkness desynchronization was higher than 2 (Figure 5A). The index values were 2.75 ± 0.02 h for women and 2.93 ± 0.02 h (*p* < 0.0001) for men, indicating some degree of desynchronization between bed and natural darkness timings. The other averaged indices were each below 1 when grouped by gender, with men showing a slightly higher bed-work desynchronization index (0.80 ± 0.01 h) than women (0.77 ± 0.01 h) (*p* < 0.0001) (Figure 5B). Lower values were found for work-natural darkness desynchronization, with women having lower levels (0.10 ± 0.01 h) than men (0.12 ± 0.01 h) (*p* = 0.0082) (Figure 5C). The integrative parameter “the Three Times Score” was 0.10 ± 0.00 for women and 0.11 ± 0.00 for men (Figure 5D) (*p* < 0.0001), in both cases indicating no relevant desynchronization of the three times.

Despite statistical significance, the differences by gender resulted in all calculated effect sizes being lower than 0.2, with the largest being 0.142 for time in bed on free days (0.126 on work days) and the smallest for wake-up time on work days (0.007).

### 3.2 Age dimension

Considering that age is a factor intimately related to rhythmicity in humans, we categorized participants into different groups based on their ages (Table 1). For further analyses, we have excluded the 5–12-year-old age group due to its small sample size and the potential for bias, given the likelihood that an adult entered the data on their behalf.

**TABLE 1:** Distribution among different age groups.

Age group	N	%
5–12 y/o	14	0.14
13–17 y/o	732	7.35
18–30 y/o	3249	32.62
31–40 y/o	2607	26.17
41–50 y/o	2242	22.51
51–64 y/o	1057	10.61
65–80 y/o	60	0.60

**FIGURE 6:**
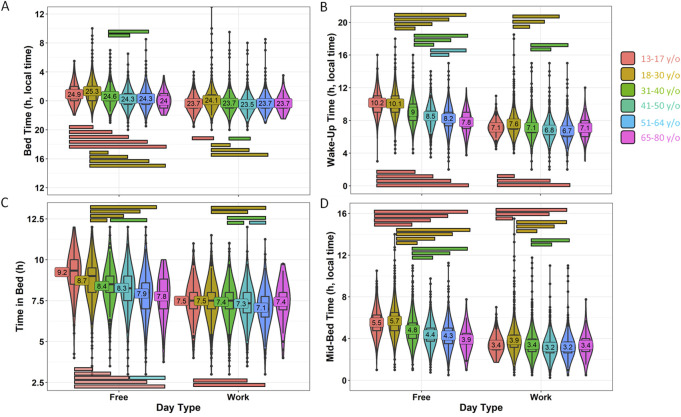
Sleep habits during workdays and free days divided by age. **(A)** Bed time, **(B)** wake-up time, **(C)** time in bed and **(D)** mid-bedtime. Violin plots represent the kernel density estimation, with median, first and third quartiles represented in box plots. The mean is indicated as a number in decimal format. The Kruskal-Wallis test was performed to compare age groups; significant differences among them (*p* < 0.002) are indicated by color-coded horizontal bars. The Wilcoxon test was used to compare work vs. free days within each variable (*p* < 0.0001).

With regard to sleep-related habits (Figure 6; Supplementary Table S3), all age groups reported differences between work and free days (*p* < 0.002). On work days, younger adult participants reported going to bed later than older adults, with adolescents falling somewhere in between. However, all groups exhibited a range of only 64 min, from 23:31 ± 0:01 for 41–50 y/o to 00:06 ± 00:01 for 18–30 y/o (Figure 6A). The calculated effect sizes were all below 0.3, which is considered small.

Reported wake-up times on work days varied within 55 min, from 6:44 ± 00:01 for 51–64 to 7:36 ± 00:01 for youngest adults (18–30 years old) (Figure 6B). In this case, the effect was moderate for both groups (18–30 vs. 51–64 years old, 0.318; 18–30 and 41–50 years old, 0.332).

However, when considering free days, both reported a bed time and wake-up time that exhibited a wider range, from 00:01 ± 00:08 (oldest) to 01:19 ± 00:01 (18–30 y/o) and from 07:48 ± 00:11 (oldest group) to 10:09 ± 00:03 (adolescents from 13 to 17 years of age), respectively (Figures 5A, B) (*p* < 0.001). In this case, the effect size on bed time was moderate between the youngest adults vs. 41–50 y/o (0.386) and vs. 51–64 y/o (0.320) groups. However, the effect size was larger when considering wake-up time on free days, with values of as much as 0.582 (large) when comparing adolescents to 51–64 years of age. The adolescent group (later timings) showed moderate differences when compared to the other age groups (earlier timings, 0.33–0.47), except with the youngest adults (18–30 y/o). Young adults also presented moderate differences when compared to the older groups (ranging from 0.338 to 0.486), except when compared to the oldest group (0.173).

According to these reported data, the time this population spent in bed (Figure 6C; Supplementary Table S3) also differed among the age groups to a greater extent on free days than when specifically examining work days, with a minimum of 7.07 ± 0.03 h for the 51–64 y/o group and 7.49 ± 0.02 for young adults on work days, showing less than 30 min of difference among adults (*p* < 0.003). The effect size was small. However, if we consider free days, the differences between the youngest and oldest age groups became greater, from 8.72 ± 0.02 h for young adults to 7.77 ± 0.17 h for elderly persons aged 65–80 (*p* < 0.0001). In this case, the effect size was also moderate between adolescents and middle-aged persons (31–40, 0.311; 41–50, 0.354; 51–64, 0.498). When correlating age with reported bed and wake-up times, we obtained negative R-values, with the strongest correlation occurring between wake-up time on free days (R = −0.449, *p* < 0.0001), followed by bed time on free days (R = −0.287, *p* < 0.0001), wake-up on work days (R = −0.236, *p* < 0.0001) and bed time on work days (R = −0.142, *p* < 0.0001) (Supplementary Figure S1).

The mid-bedtime (Figure 6D; Supplementary Table S3) exhibited similar results as bed time and wake-up time. In this case, the earliest timing corresponded to adults (41–64) (3:12 ± 0:07) while the latest occurred in the youngest adults (3:51 ± 0:01) on work days, with a range of around 40 min (*p* < 0.0001). Only a moderate effect was found between the youngest adults and the 41–50-year-old group (0.337). Again, on free days, this timing marker exhibited values within wider limits, with 3:54 ± 0:08 (for the elderly) and 5:42 ± 0:01 for the youngest adults, around 2 h of difference (*p* < 0.0001). In this case, some moderate effect sizes were found (e.g., adolescents vs. 41–50 y/o (0.404), 51–64 y/o (0.479), 65–80 y/o (0.324); or young adults vs. 31–40 y/o (0.327), 41–50 y/o (0.482), 51–64 y/o (0.437)).

**FIGURE 7:**
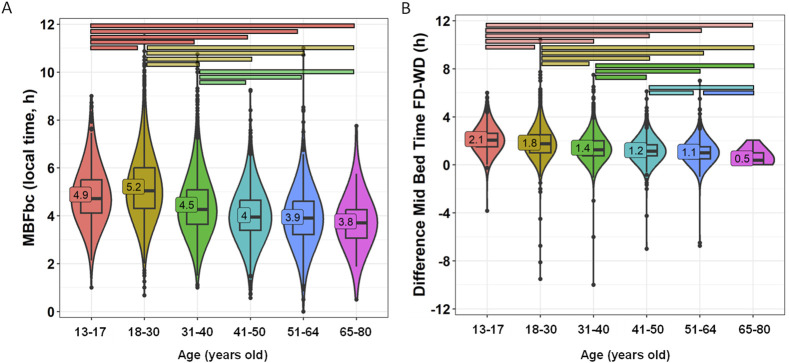
MBFbc **(A)** and difference in mid-bedtime between free and work days **(B)** divided by age groups. Violin plots represent the kernel density estimation, with median, first and third quartiles represented in box plots. The mean is indicated as a number in decimal format. The Kruskal-Wallis test was performed to compare age groups; significant differences among them are indicated by color-coded horizontal bars. Significant differences between genders are indicated by horizontal bars (*p* < 0.0001).

MBFbc, a modified version of MSFsc (Figure 7A), ranged from 03:45 ± 00:08 in the oldest adult group to 05:13 ± 00:01 in the youngest adult group (18–30 years old) (*p* < 0.0001). Some moderate effect sizes were found (e.g., adolescents vs. 41–50 y/o, 0.332; and vs. 51–64 y/o, 0.394; and young adults vs. 31–40 y/o, 0.305; 41–50 y/o, 0.466; 51–64 y/o, 0.417).

Again, we calculated the difference in mid-bedtimes between free and work days (similar to social jet lag) by age group (Figure 7B; Supplementary Table S4), revealing a distinct pattern of greater social jet lag (relative to bed time) values in younger participants (2.14 ± 0.04 h and 1.84 ± 0.02 h for 13–17 y/o and 18–30 y/o, respectively) as compared to older groups (1.08 ± 0.03 and 0.54 ± 0.07 h for 51–64 y/o and 65–80 y/o, respectively) (*p* < 0.0001). Between these extremes, intermediate values with a clear detrimental pattern can be observed (Figure 7B). In this case, several moderate effect sizes were found between adolescents vs. older groups, with a notably large difference (0.536) between adolescents and the 51–64-year-old group.

Both MBFbc and this difference are strongly correlated (R = 0.565, *p* < 0.0001, Supplementary Figure S1). When calculating the percentage of participants with more than 2 h of difference between free and work days, we also found this detrimental pattern, ranging from 50.41% for 13–17 y/o to 1.67% for 65–80 y/o (Supplementary Table S4).

**FIGURE 8:**
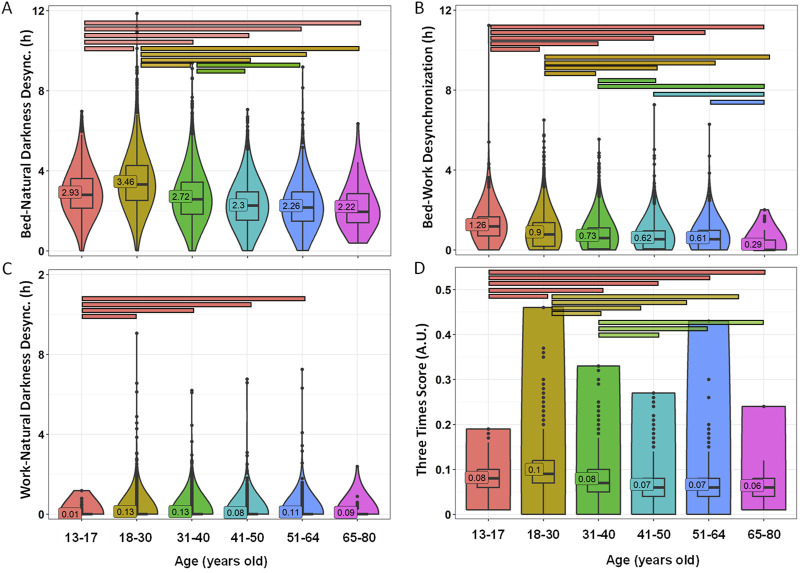
Desynchronization indexes by age groups. Bed-natural darkness **(A)**, bed-work **(B)**, work-natural darkness **(C)** desynchronizations and Three Times Score **(D)**. Violin plots represent the kernel density estimation, with median, first and third quartiles represented in box plots. The mean is indicated as a number in decimal format. The Kruskal-Wallis test was performed to compare age groups; significant differences among age groups (*p* < 0.0013) are indicated by color-coded horizontal bars.

Desynchronization between bed, natural darkness and work timings was also calculated for each age group (Figure 8; Supplementary Table S4). Again, only bed-natural darkness desynchronization was higher than 2, indicating relevant desynchronization between bed and natural darkness time frames. Lower values were found in the youngest groups (3.46 ± 0.02 in 18–30 y/o group and 2.93 ± 0.04 in the 13–17 y/o group) as compared to the older groups (2.22 ± 0.15 for 65–80 y/o) (*p* < 0.0001), with a decreasing pattern towards the older ages (Figure 8A). The effect size was moderate for 18–30 y/o vs. 41–50 y/o (0.422) and 51–64 y/o (0.378). Bed-work desynchronization also showed a clear decline with age, ranging from 1.26 ± 0.03 in the adolescent group (moderate desynchronization) to 0.29 ± 0.06 (insignificant desynchronization) in the elderly group (*p* < 0.0001). The effect sizes were moderate for children vs. the elderly (0.464) and for adolescents vs. 31–40 y/o (0.300), 41–50 y/o (0.376), 51–64 y/o (0.434) and 65–80 y/o (0.343) (Figure 8B). The work-natural darkness desynchronization index averaged across all age groups showed low values, all below 0.13, indicating insignificant desynchronization between social and environmental timing (Figure 8C). The averaged Three Times Score showed a similar pattern, with maximum values for adolescents and young adults (0.12) as compared to older populations (0.07 – 0.10) (*p* < 0.0001) (Figure 8D). The differences were moderate between adolescents vs. 41–50 y/o (0.300) and 51–64 y/o (0.350); and between young adults vs. the 41–50 y/o (0.401) and 51–64 y/o groups (0.356).

### 3.3 Season and daylight saving time

**FIGURE 9:**
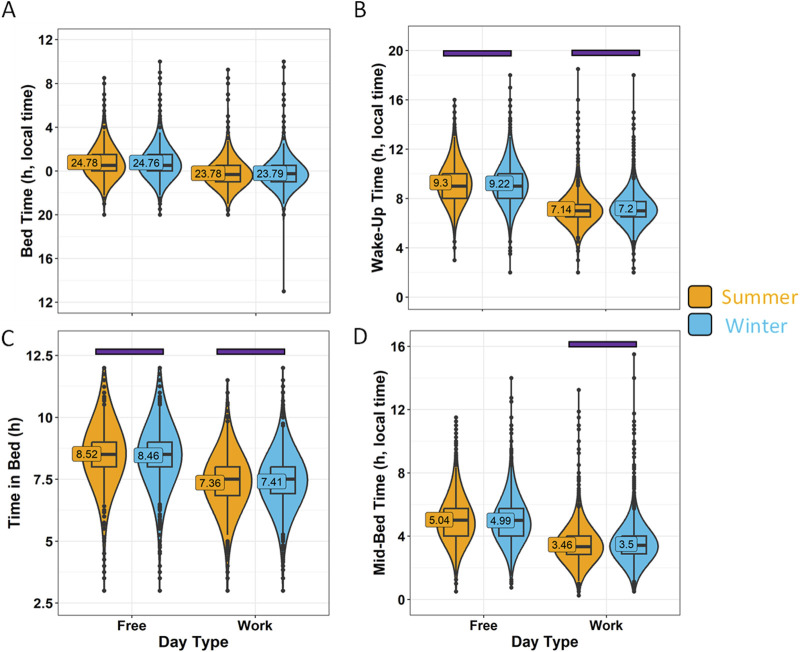
Sleep habits on work days and free days divided by season. **(A)** Bed time, **(B)** wake-up time, **(C)** time in bed and **(D)** mid-bedtime in summer (orange) and winter (blue). Violin plots represent the kernel density estimation, with median, first and third quartiles represented in box plots. The mean is indicated as a number in decimal format. The Mann-Whitney U test was performed to compare both seasons; differences within each day type within the same season are indicated by horizontal purple bars (*p* < 0.05).

The responses obtained in January-March and October-December were considered “Winter” (6125, 61.5%), while responses in April, May, June, July, August and September, were considered “Summer” (3838, 38.5%). The reported bed time (Figure 9A) did not differ between seasons (*p* > 0.05). However, the wake-up time (Figure 9B) during the winter was around 5 min earlier on free days (*p* = 0.0192), and later on work days (*p* = 0.0002). According to these reported data, participants responding in the summer would have spent more time in bed than in the winter on free days (*p* = 0.0135) and less time in bed on work days (*p* = 0.025) (Figure 9C). However, these differences reflect less than 1% of the total time in bed. The mid-bedtime timing marker (Figure 9D) occurred only 3 min later in the winter than in the summer on work days (*p* = 0.0038), with no differences on free days between the two seasons (*p* > 0.05). The calculated effect sizes in this case were all of small magnitude.

**FIGURE 10:**
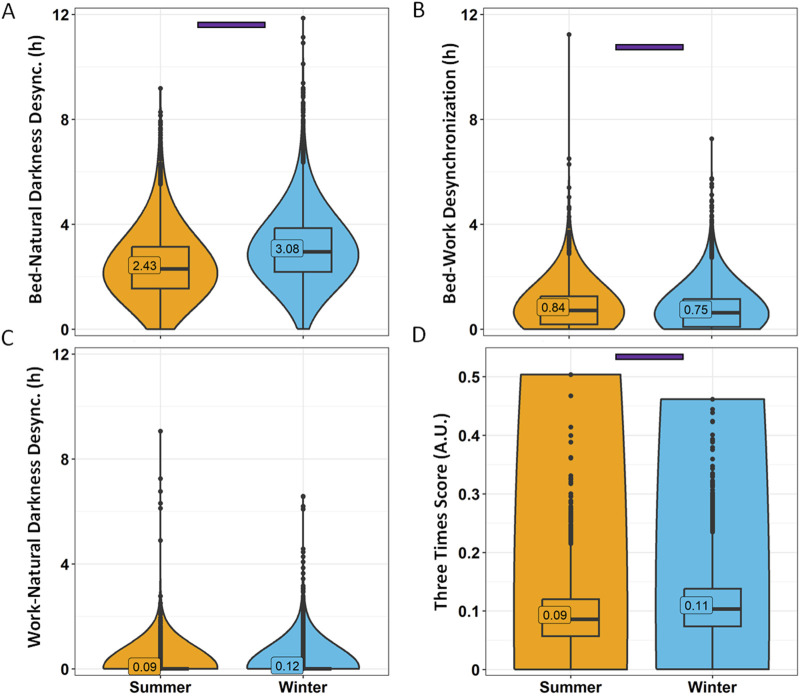
Desynchronization indexes by season. Bed-natural darkness **(A)**, bed-work **(B)**, work-natural darkness **(C)** desynchronizations and Three Times Score **(D)**. Violin plots represent the kernel density estimation, with median, first and third quartiles represented in box plots. The mean is indicated as a number in decimal format. The Kruskal-Wallis test was performed to compare age groups; significant differences among age groups (*p* < 0.0013) are indicated by color-coded horizontal bars.

When considering desynchronization indexes (Figure 10), bed-natural darkness desynchronization was greater in Winter (3.08 ± 0.02 h) than in Summer (2.43 ± 0.02 h) (*p* < 0.0001) (Figure 10A). Bed-work desynchronization, however, was greater in summer (0.84 ± 0.01 h) than in winter (0.75 ± 0.01 h) (Figure 10B), although, as previously stated, it can be considered irrelevant (<1 h). The three times score was higher in winter (0.11) than in summer (0.09) (Figure 10D).

**FIGURE 11:**
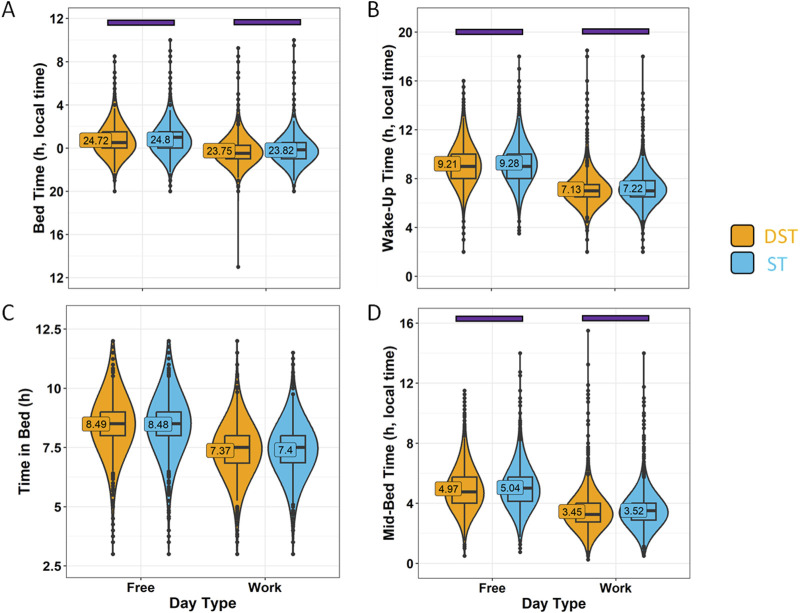
Sleep habits on work days and free days divided by daylight-saving time (DST) or standard time (ST). **(A)** Bed time, **(B)** wake-up time, **(C)** time in bed and **(D)** mid-bedtime in summer (orange) and winter (blue). Violin plots represent the kernel density estimation, with median, first and third quartiles represented in box plots. The mean is indicated as a number in decimal format. The Mann-Whitney U test was performed to compare both seasons; differences within each day type within the same season are indicated by horizontal purple bars (*p* < 0.05).

**FIGURE 12:**
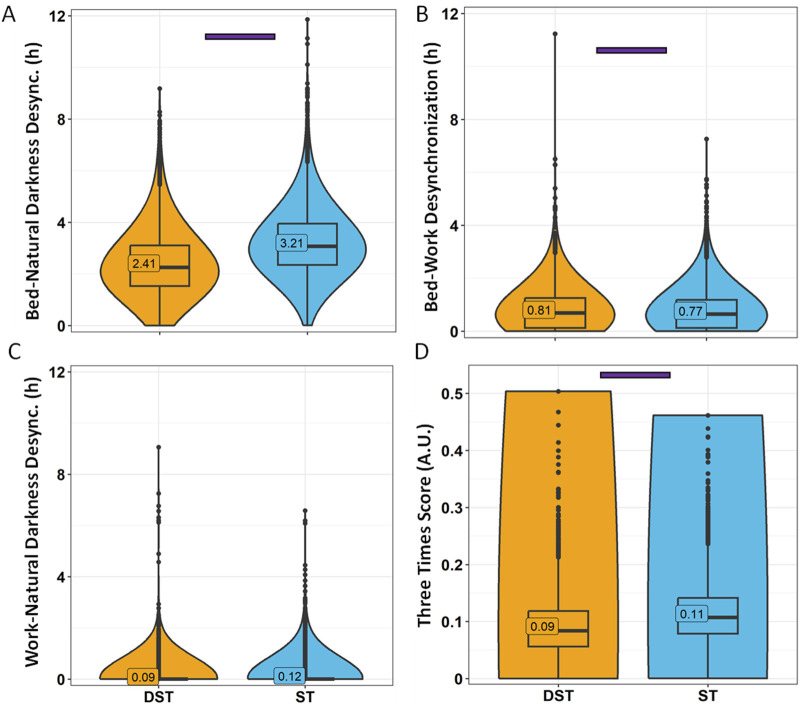
Desynchronization indexes by daylight-saving time (DST) or standard time (ST). Bed-natural darkness **(A)**, bed-work **(B)**, work-natural darkness **(C)** desynchronizations and Three Times Score **(D)**. Violin plots represent the kernel density estimation, with median, first and third quartiles represented in box plots. The mean is indicated as a number in decimal format. The Kruskal-Wallis test was performed to compare age groups; significant differences among age groups (*p* < 0.0013) are indicated by color-coded horizontal bars.

Considering the association between season and standard time or daylight saving time, we also analysed the data considering the official timing when each response was received, i.e., daylight saving time (DST, in general, from November to March) or standard time (ST, in general, from April to October) (Figure 11). In this case, although the dissimilarities in sleep-related habits were also minimal (with no differences in time in bed), the disparities in bed-natural darkness desynchronization were potentiated with respect to season (Figure 12), increasing the differences between ST (3.21 ± 0.02 h) and DST (2.41 ± 0.02 h) (effect size = 0.319, moderate). Considering the strong association between both variables (official timing and season), we included them as covariates, obtaining a strongest effect for official timing (*p* < 0.0001) than that for season (*p* = 0.0107). The three times scores were similar to those found for winter (ST) vs. summer (DST).

### 3.4 Regression analysis

A mixed-effect regression analysis (with participant identification as random effect) was conducted to assess whether the biological, social and environmental factors considered in the study significantly predict the sleep-related parameters, including bedtime, wake-up time, time in bed and mid-bedtime. The independent variables encompassed biological factors, such as age and gender (dichotomous, male/female); social related factors, such as work start and end times and day type (dichotomous, free day/work day), as well as use of alarm clock (dichotomous, Y/N). For environmental factors, considering the multicollinearity due to the correlation between sunrise, sunset and official time (DST/ST) or season, and after applying Akaike information criterion, only sunset time was included in the model.

The fitted regression models are as shown in Tables 2–5, where positive coefficients indicate later timings, while negative coefficients represent earlier timings:

**TABLE 2:** Coefficients for bed time.

	Estimate (β)	Standard error	T Value	Pr (>|t|)
Intercept	22.4144616	0.2160384	103.752	
Day type (WD)	−0.9118506	0.0154273	−59.106	<2.2e-16
Work start time (h)	0.1997113	0.0058039	34.410	<2.2e-16
Work end time (h)	0.0031239	0.0040538	0.771	0.4409
Alarm clock usage (Yes)	−0.1039529	0.0180572	−5.757	8.568e-09
Age (year)	−0.0212709	0.0008845	−24.047	<2.2e-16
Gender (men)	0.3473451	0.0213046	16.304	<2.2e-16
Sunset time (h)	0.0557868	0.0103448	5.393	6.49e-08

**TABLE 3:** Coefficients for wake up time.

	Estimate (β)	Standard error	T Value	Pr (>|t|)
Intercept	7.6253197	0.2041169	37.358	
Day type (WD)	−1.8508299	0.0198301	−93.334	<2.2e-16
Work start time (h)	0.2683064	0.0054800	48.961	<2.2e-16
Work end time (h)	0.0080555	0.0038279	2.104	0.03534
Alarm clock usage (Yes)	−0.3427512	0.0219618	−15.607	<2.2e-16
Age (year)	−0.0380694	0.0008383	−45.410	2.699e-09
Gender (men)	0.1196623	0.0201149	5.949	4.01E-11
Sunset time (h)	0.0199459	0.0097665	2.042	0.04112

**TABLE 4:** Coefficients for time in bed.

	Estimate (β)	Standard error	T Value	Pr (>|t|)
Intercept	9.2641588	0.1778633	52.086	
Day type (WD)	−0.8613106	0.0173614	−49.611	<2.2e-16
Work start time (h)	0.0628613	0.0047751	13.164	<2.2e-16
Work end time (h)	0.0083555	0.0033355	2.505	0.01224
Alarm clock usage (Yes)	−0.3624247	0.0192046	−18.872	<2.2e-16
Age (year)	−0.0175093	0.0007306	−23.967	<2.2e-16
Gender (men)	−0.2349314	−0.2349314	−13.404	<2.2e-16
Sunset time (h)	−0.0362501	0.0085102	−4.260	2.048e-05

**TABLE 5:** Coefficients for mid-bed time.

	Estimate (β)	Standard error	T Value	Pr (>|t|)
Intercept	3.043650	0.190992	15.936	
Day type (WD)	−1.350671	0.015509	−87.092	<2.2e-16
Work start time (h)	0.236000	0.005130	46.005	<2.2e-16
Work end time (h)	0.004510	0.003583	1.259	0.01224
Alarm clock usage (Yes)	−0.267955	0.017822	−15.035	<2.2e-16
Age (year)	−0.029822	0.000783	−38.086	<2.2e-16
Gender (men)	0.234427	0.018832	12.448	<2.2e-16
Sunset time (h)	0.037346	0.009143	4.085	2.048e-05

Among the dichotomous independent variables, Day Type (work day) significantly contributed to predicting sleep-related variables, especially Wake-up Time (β = - 1.85, *p* < 2.2e-16). Work Start Time also significantly predicted Bed time (β = 0.20) and Wake-up Time (β = 0.27) (*p* < 2.2e-16). The alarm clock usage also significantly predicted earlier wake-up times (β = −0.34, *p* < 2.2e-16). Gender significantly predicted the dependent variables analyzed, especially Bed time (β = 0.35, *p* < 2.2e-16), indicating that being a man may involve going to bed up to 20 min later. Age also significantly predicted sleep related variables, indicating that someone 50 years older would have an advancement in sleep-related habits of around 1 h. Sunset time also had some contribution to the bed schedule regarding bed time (β = 0.06, *p* < 6.49e-08).

## 4 Discussion

In this work, we explored different sleep/bed-related habits and work-related schedules in a large population of Spanish participants, considering both free and work days. We have also included age as a relevant biological factor, and gender, assuming gender and sex match in most cases, to consider in terms of sleep-related habits and rhythmicity.

The differences between work and free days found in sleep-related habits were as expected, with a mid-bedtime (similar, although not equivalent, to the mid-sleep phase (Roenneberg et al., 2003)) around 4–5 a.m., which is consistent with previous results (Roenneberg et al., 2007). This marker occurred around 1.5 h later on free days with respect to work days, considering the entire population. This is consistent with previous publications, which have reported that a relevant portion of the population experiences a variance in the timing of this phase marker between free and workdays, ranging from 1 h to over 2 h (Roenneberg et al., 2003; 2012; Wittmann et al., 2006). However, our results show a greater influence of sleep offset (or wake-up) rather than bed time, in terms of both differences in mid-bedtime and time in bed, which is in contrast with the results reported in pre-industrial societies (Yetish et al., 2015). This is probably due to the clear effect of social duties related to work on our participants. In addition to day type, we also included work start and end times as social factors that could influence sleep-related habits. In this regard, with our regression analyses, we found that a 1-h difference in the work start time could lead to more than a 15-min difference in wake-up time, also influencing the central bed timing, but with only a small influence on time in bed (4 min per hour of delay in start time). Previous studies, however, have found that time in bed and sleep duration can be significantly increased per hour of delay in start time in adolescents (Alfonsi et al., 2020; Widome et al., 2020; Edinborough et al., 2023) and adults (Lombardi et al., 2014).

Regarding differences by gender in terms of sleep-related habits, in our sample, women preferred to go to bed earlier than men both on work and on free days. On free days, women also reported that they woke up later than men. Thus, apparently, women were in bed between 15–20 min longer than men on both day types, even when incorporating age as a covariate. Furthermore, the regression analysis performed indicated that being a man may delay bed time by around 20 min. These results are consistent with previously reported data on gender sleep differences in the general population (Tonetti et al., 2008; Jonasdottir et al., 2021) and in teachers (de Souza et al., 2018). In the latter study, however, differences by gender disappeared when the analysis was adjusted according to shifts or levels of education. It has also been previously reported that women tend to claim to need longer durations of sleep under 55 years of age (Tonetti et al., 2008). However, in a recent actigraphic study with 68,604 Japanese residents, women showed shorter total sleep durations than men of similar age, especially after 30 years of age, as well as greater reductions in sleep efficiency (Li et al., 2021). In this sense, we should highlight that our results are based on reported bed times (we did not ask about sleep latency) rather than on objective measures of sleep, so we could not actually claim that the women in our sample slept more than the men.

In any case, these gender differences in sleep timing have been previously interpreted as a product of socio-cultural influences (Park et al., 1997) or biological factors, including sex hormones (Carskadon et al., 1993; Roenneberg et al., 2004; Tonetti et al., 2008). Although we cannot rule out possible biological effects, in this study we have also explored the differences between men and women in terms of work schedules and work-related social habits. First, we found that women start work 12 min later than men on average. Also, one revealing aspect was the distribution of work end times, with most women engaging in intensive or half-days, while men in this sample tended to work split shifts. Both results could be related to the fact that, according to the Organization for Economic Cooperation and Development, men in Spain dedicate more hours to remunerated work than women (Organisation for Economic Co-operation and Development, 2022), while the latter invest more time in caregiving (Casado-Mejía and Ruiz-Arias, 2016; Fernández Torralbo et al., 2020). Indeed, this could be also in agreement with our results on time to get ready from home to work, which is longer for women, as it may be related to family care.

When considering different age groups, the greatest differences between free and work days (equivalent to social jetlag) were seen in the youngest groups, with mid-bedtimes occurring nearly 2 h later on free days. These results agree with previous data published by Roenneberg et al. (2003), with 21–30-year-old adults showing a 2 h later midsleep phase on free days with respect to work days. In our study, these differences were attenuated in older groups, with only 30 min of variation in the 65–80-year-old group, which is also consistent with these data (Roenneberg et al., 2003). This consistency, however, is rather surprising considering the difference in geographical area and country (Germany vs. Spain) and also the official time adopted in Spain, which does not coincide with its solar time (Bonmatí-Carrión et al., 2022). In this sense, however, comparing our modified MBFbc parameter to the data obtained by Roenneberg et al. (2019a) suggests that our population may be shifted towards later chronotypes, and presumably does not cover the entire range of chronotypes previously described in Roenneberg’s study (Roenneberg et al., 2019a). This could be an effect of the mismatched official and solar time (Bonmatí-Carrión et al., 2022) in Spain. We could also explain these results in terms of the possible selection bias that our design involves, one of the limitations stated at the end of this manuscript. We did find a correlation between chronotype and differences between free and work days, which is also reflected in the detrimental percentages of participants with social jet lag as age increases (reviewed in (Taillard et al., 2021).

Regarding sleep habits *per se*, both bed and wake-up times play a part in these differences in mid-bedtime. However, it should be noted that the greatest contribution is made by the wake-up time, which occurs 2.5 h later in the youngest groups on free days with respect to work days, while bed time is more homogenous among the age groups and day types. This is also consistent with previous results based on objective measures in the general population (Jonasdottir et al., 2021), and may reflect that the social clock is waking people up too early on workdays, which also shortens their time in bed (and thus their sleep duration) on those days. On free days, however, the biological clock is free to follow its natural phase, and is especially delayed in younger groups. This is also in agreement with recent results obtained by our group in an elderly population (Bonmatí-Carrión et al., 2022).

Regarding the desynchronization among the three time frames (bed, natural darkness and work schedule), we did not detect any relevant desynchronizations between work and natural darkness timing. The desynchronization between bed and work times was moderate (above 1) only in the adolescent group. Although in our study we cannot properly assess chronotype or internal time, this would be in accordance with the delayed chronotype widely described in this age group, together with their early school starting times (Fischer et al., 2017). The bed-natural darkness desynchronization, however, was greater than 2 in all age groups, with an increasingly detrimental pattern towards older ages, with adolescents and young adults standing out with values close to or greater than 3.

With regard to bed-work desynchronization, this showed a clear decline with age. In this sense, the fact that a chronotype advance with age (Fischer et al., 2017) would favor better bed-work synchronizations in a context where a morning work schedule is generalized. Wittmann et al. (2006) already showed that later chronotypes show the largest differences in sleep timing between work and free days, probably due to a considerable sleep debt on work days, for which they try to compensate on free days (Wittmann et al., 2006). Again, we cannot directly assess internal time, so our results are just a proxy for the possible effect of chronotype.

Our results barely showed any differences between seasons in terms of sleep, which is in accordance with previous studies even in subarctic regions where the variations in photoperiod are huge (Johnsen et al., 2013). Although a clear seasonal effect has been previously reported in preindustrial (Yetish et al., 2015) and industrialized societies (Stothard et al., 2017), we should bear in mind that those studies involve natural or imposed exposure to a natural light-dark cycle over the seasons. Our study, however, is based on self-reported information about habitual routines (and not necessarily pertaining to the season when the questionnaire was answered), and it is not an objective controlled study. Furthermore, our study is not longitudinal, so comparisons among the participants are not possible. These reasons, among others, could explain the absence of differences between seasons. In addition, when we separated the data according to the time at the moment of response (Daylight Saving Time or Standard Time), we observed that bed-natural darkness desynchronization was greater in Standard Time (ST) compared to Daylight Saving Time (DST), contrary to our initial expectations. This apparent discrepancy may stem from the prevalence of late chronotypes and social habits in Spain, where people tend to delay their bedtime in relation to natural darkness. By artificially delaying the midpoint of natural darkness (which happens during DST), it would apparently better align with the mid-bedtime. In this sense, we should take into account the separation of sleep-related and social habits from the natural photoperiod, since the natural darkness daily period is usually longer and more advanced compared to the human rest period. Consequently, actual personal light exposure does not necessarily correspond to the natural photoperiod. In this sense, we did previously find greater bed-natural darkness desynchronizations in the western region of Spain (which has a mismatched official time considering its geographical location) than in Portugal (where official time matches the geographical location during the standard time period), but in that case, personal light exposure and distal body temperature were measured directly and objectively through wearable devices (Bonmatí-Carrión et al., 2022). We must acknowledge that in our study we are assessing bed time rather than sleep onset, which can be very much influenced by social schedules. However, in our experience with volunteers, bedtimes and wake-up times may be easier to recall and self-report than sleep onset time or sleep latency, making it easier for participants to complete the entire questionnaire. Also, in a normal population, average sleep latency has been established at around 11.7–11.8 min (Iskander et al., 2023) and other studies found sleep latencies around 12–17 min (Juda et al., 2013). Therefore, assuming a normal population, sleep latency should not produce significantly different results.

In this line, in our regression analysis, we identified a significant contribution of sunset time in predicting bed timing. Each hour of difference in sunset was associated with an approximately 2-min influence in the midpoint of bedtime. Although significant, this contribution could be small due to the fact that the participants’ lives might be strongly dominated by social duties, which are importantly masking the natural light/dark cycle. The ubiquitousness of over-illuminated nights, due to the use of artificial light, and under-illuminated days could be influencing this lack of link between natural light-dark cycle and human sleep patterns. In this sense, in a previous study, we found that artificial light could be “compensating” for differences in natural light, for example, in daylight saving time transitions (Arguelles-Prieto et al., 2022). In our current study, social factors as day type, work start time and alarm clock usage showed greater influence in sleep related habits than natural environmental factors such as sunset, sunrise or season. However, there is previous evidence that suggests that sleep patterns on free days are closely related to the solar schedule, which is related to geographical location (Porcheret et al., 2018). Other studies have found a relationship between the incidence of cancer and western geographical locations within a time zone, which has been related to the possible greater social jetlag in those areas (Borisenkov, 2011; Gu et al., 2017; Caporaso et al., 2018). In this sense, a previous study found that after exposure to only natural light, the internal circadian clock synchronizes to solar time in such a way that the beginning of the internal biological night occurs at sunset, and the end of the internal biological night occurs before wake-up time (just after sunrise) (Wright et al., 2013). In this sense, again, we must highlight the fact that we are not assessing sleep onset but bed time, which can be influenced by social schedules.

This study has certain limitations related to the interpretation of the results, due to the manner in which the data were collected. First, the sample was not randomized or stratified, and a bias may exist since participants voluntarily approaching this test may have a prior specific interest in the topic. Additionally, no previous analyses on sample size were conducted, and the data collection was arbitrarily concluded upon reaching a certain number of responses. Second, all variables in this study were subjective, and are based on recall. In addition, the questionnaire was self-administered without supervision, which could have led to errors, despite the strict filtering process. Third, we did not ask about sleep latency, so we consider only the time when participants usually go to bed and when they usually wake-up. This makes it impossible to differentiate between time in bed and actual sleep time. Also, wake up time does not always coincide with getting up time, so time in bed needs to be also considered as an approximation of actual time in bed. However, in our previous experience, it is easier for volunteers to recall the time they wake up rather than the time they get up. Similarly, the inability to derive a reliable circadian phase marker from our data makes it challenging to draw precise conclusions regarding circadian alignment. Fourth, we did not ask about work type, or pay any special attention to shift-workers. Furthermore, questions on light exposure would have been desirable. Also, our biological-social interplay is only related to social and biological time frames. It would have been desirable to include further covariates, such as education level, marital status, children, caregiving tasks/obligations, other social habits, such as meal timing, hobbies or health related aspects, among others. Finally, the questionnaire has not been compared to previous questionnaires or validated through internal testing. However, it was not intended to replace previously validated questionnaires. We propose it solely as a complementary tool to explore the interaction between sleep-related habits, work schedules, and the natural photoperiod.

Our study provides insights into sleep-related habits and their interactions with social and environmental cues in a large Spanish population. This opens the way to future improved versions of the questionnaire used, including additional questions on bed-related habits, natural photoperiod and work-related timings, as well as including other important daily habits, such as meal times, or other social related habits, and also expansion to other countries. Future versions of this tool will also be validated against widely established circadian assessment tools. Understanding these time frame interactions will be essential for developing tailored interventions to improve sleep and wellbeing in different population groups.

## Data Availability

The raw data supporting the conclusions of this article will be made available by the authors upon request, without undue reservation.
