# Supplementary material for: The interplay among sleep patterns, social habits, and environmental cues: insights from the Spanish population and implications for aligning daily rhythms
Source: Front Physiol. 2024 Oct 25;15:1323127. doi: 10.3389/fphys.2024.1323127 (PMC11543482; doi:10.3389/fphys.2024.1323127)
Supplement: Supplementary file 1 [file DataSheet1.pdf]

Supplementary Material

The Interplay between Sleep Patterns, Social Habits, and Environmental Cues: Insights from the Spanish Population and implications on circadian health

María-Ángeles Bonmatí-Carrión *, Jesús Vicente-Martínez, Juan Antonio Madrid, Maria-Angeles Rol

*** Correspondence: María-Ángeles Bonmatí-Carrión: mabonmati@um.es**

1 Supplementary Figures and Tables

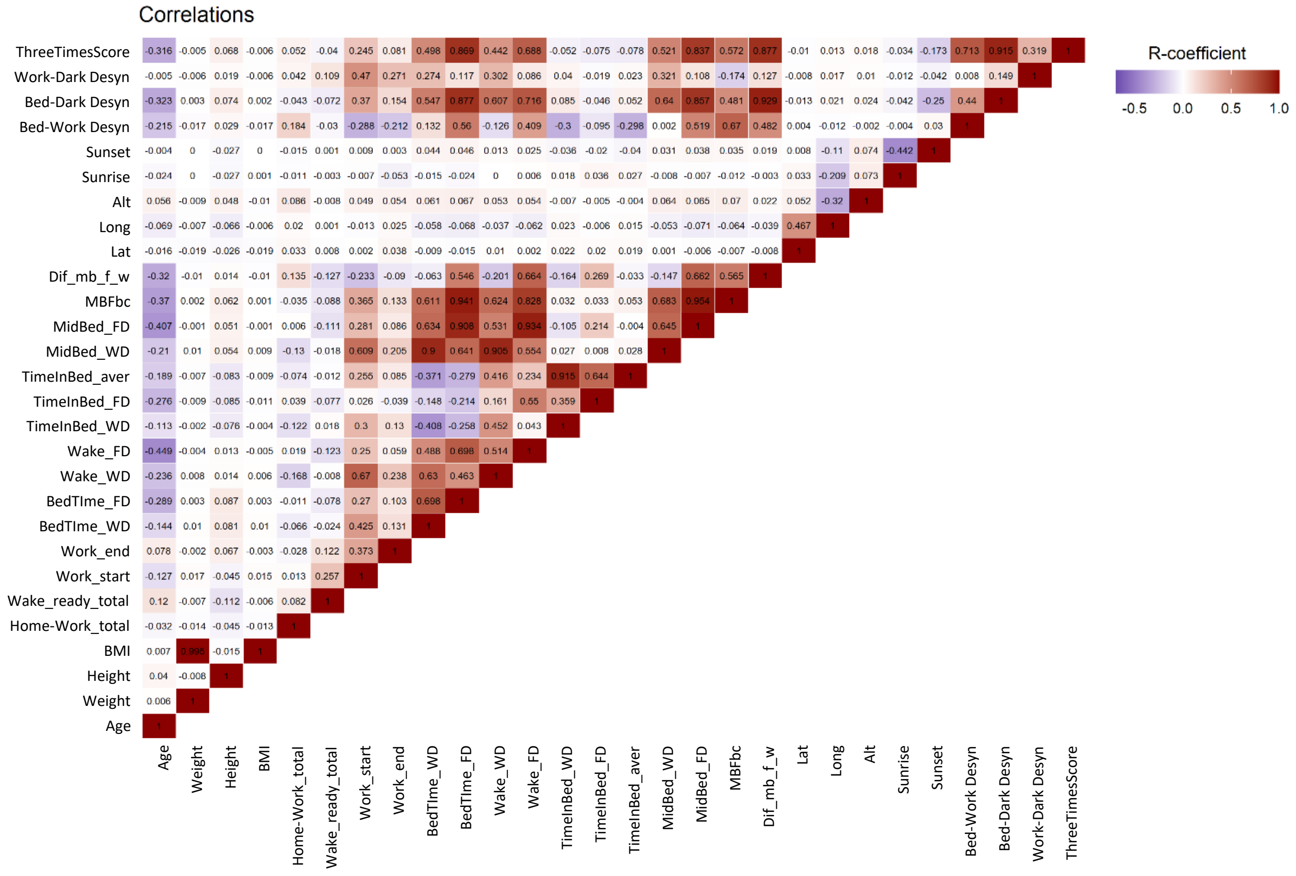

Figure S1. Spearman's correlation heatmap with R-coefficient values indicated in each cell. BMI: Body Mass Index (kg/m^2); wd: work day; fd: free day; MBFbc: modified version of the sleep corrected midsleep in free days (MSFsc); FD: free day; WD: work day. Dif_mb_f_w: difference in bed time free day – work day; Lat: latitude; Long: longitude; Alt: altitude; Bed-Work Desyn: Bed-

Work desynchronization; Bed-Dark Desyn: Bed-Natural Darkness desynchronization; Work-Dark Desyn:: Work-Natural Darkness desynchronization.

Table S1. Sleep habits during work days and free days split by gender. Values are represented as mean ± SEM, both in decimal and hh:mm format, where appropriate.

	Women						Men					
	Free day			Work day			Free day			Work day		
Bed Onset Time (h)	24.65	±	0.02	23.68	±	0.02	24.91	±	0.02	23.92	±	0.02
(hh:mm)	00:39	±	00:01	23:40	±	0:01	00:54	±	00:01	23:55	±	00:01
Wake-Up Time (h)	9.28	±	0.02	7.18	±	0.02	9.21	±	0.03	7.17	±	0.02
(hh:mm)	09:16	±	0:01	07:10	±	0:01	09:12	±	0:01	07:10	±	0:01
Time in Bed (h)	8.63	±	0.02	7.50	±	0.01	8.30	±	0.02	7.25	±	0.01
(hh:mm)	8:37	±	0:01	07:30		00:00	8:18	±	00:01	07:15	±	00:00
Mid-Bed Time (h)	4.97	±	0.02	3.43	±	0.01	5.06	±	0.02	3.54	±	0.02
(hh:mm)	04:58	±	0:01	03:25	±	0:01	05:03	±	0:01	03:32	±	0:01

Table S2. Other sleep parameters and social habits related to work schedules split by gender. Desynchronization indexes and three times score. Values are represented as mean ± SEM, both in decimal and hh:mm format, where appropriate.

	Women			Men		
BMI	22.95	±	0.06	25.02 ± 0.06		
Averaged Time in Bed (h)	7.81	±	0.01	7.54	±	0.01
(hh:mm)	7:48	±	0:00	7:32	±	0:00
MBFbc (h)	4.53	±	0.02	4.65	±	0.02
(hh:mm)	04:31	±	0:01	04:39	±	0:01
Difference Mid Bed Time (FD-WD), social jet lag (h)	1.53	±	0.01	1.51	±	0.02
(hh:mm)	01:31	±	0:00	01:30	±	0:01
Commute time (min)	29.00	±	0.29	26.52		0.30
Time to get ready (min)	56.54	±	0.46	47.95		0.50
Work Start (h)	9.17	±	0.03	8.97	±	0.03
(hh:mm)	09:10	±	0:01	08:58	±	0:01
Work End (hh:mm)	16.58	±	0.04	17.04	±	0.04
(hh:mm)	16:34	±	0:02	17:02	±	0:02
Work-Natural Darkness Desynchronization (h)	0.10	±	0.01	0.12	±	0.01

Bed-Natural Darkness Desynchronization (h)	2.75	±	0.02	2.93	±	0.02
Bed-Work Desynchronization (h)	0.77	±	0.01	0.80	±	0.01
Three Times Score (A.U.)	0.10	±	0.00	0.11	±	0.00

BMI: Body Mass Index (kg/m^2); MBFbc: modified version of the sleep corrected midsleep in free days (MSFsc); FD: free day; WD: work day.

Table S3. Sleep habits during work days and free days split by age group. Values are represented as mean ± SEM, both in decimal and hh:mm format, where appropriate.

	Work Day					
	13-17 years/old	18-30 years/old	31-40 years/old	41-50 years/old	51-64 years/old	65-80 years/old
Bed Onset Time (h)	23.65 ± 0.04	24.10 ± 0.02	23.71 ± 0.02	23.52 ± 0.02	23.67 ± 0.04	23.66 ± 0.15
(hh:mm)	23:39 ± 00:02	00:06 ± 00:01	23:42 ± 00:01	23:31 ± 00:01	23:40 ± 00:02	23:39 ± 00:09
Wake-Up Time (h)	7.14 ± 0.02	7.60 ± 0.02	7.13 ± 0.02	6.84 ± 0.02	6.74 ± 0.03	7.09 ± 0.17
(hh:mm)	07:08 ± 0:01	07:36 ± 0:01	07:07 ± 0:01	06:50 ± 0:01	06:44 ± 0:01	07:05 ± 0:10
Time in Bed (h)	7.48 ± 0.04	7.49 ± 0.02	7.42 ± 0.02	7.31 ±± 0.02	7.07 ± 0.03	7.43 ±± 0.15
(hh:mm)	7:28 ± 0:02	7:29 ± 0:01	7:25 ± 0:01	7:18 0:01	7:04 ± 0:01	7:25 0:09
Mid-Bed Time (h)	3.40 ± 0.03	3.85 ± 0.02	3.42 ± 0.02	3.18 ± 0.02	3.21 ± 0.03	3.38 ± 0.14
(hh:mm)	03:24 ± 0:01	03:51 ± 0:01	03:25 ± 0:01	03:10 ± 0:01	03:12 ± 0:01	03:22 ± 0:08

	Free Day					
	13-17 years/old	18-30 years/old	31-40 years/old	41-50 years/old	51-64 years/old	65-80 years/old
Bed Onset Time (h)	24.92 ± 0.05	25.33 ± 0.03	24.64 ± 0.03	24.26 ± 0.02	24.32 ± 0.04	24.03 ± 0.14
(hh:mm)	00:55 ± 00:03	01:19 ± 00:01	00:38 ± 00:01	00:15 ± 00:01	00:19 ± 00:02	00:01 ± 00:08
Wake-Up Time (h)	10.16 ± 0.05	10.06 ± 0.03	9.04 ± 0.03	8.53 ± 0.03	8.25 ± 0.04	7.80 ± 0.19
(hh:mm)	10:09 ± 0:03	10:03 ± 0:01	09:02 ± 0:01	08:31 ± 0:01	08:15 ± 0:02	07:48 ± 0:11
Time in Bed (h)	9.24 ± 0.04	8.72 ± 0.02	8.39 ± 0.02	8.27 ± 0.02	7.93 ± 0.04	7.77 ± 0.17
(hh:mm)	9:14 ± 0:02	8:43 ± 0:01	8:23 ± 0.01	8:16 ± 0:01	7:55 ± 0:02	7:46 ± 0:10
Mid-Bed Time (h)	5.54 ± 0.05	5.70 ± 0.02	4.84 ± 0.02	4.39 ± 0.02	4.29 ± 0.04	3.91 ± 0.14
(hh:mm)	05:32 ± 0:03	05:42 ± 0:01	04:50 ± 0:01	04:23 ± 0:01	04:17 ± 0:02	03:54 ± 0:08

Table S4. Other sleep parameters and social habits related to work schedules split by age group. Desynchronization indexes and three times score. Values are represented as mean ± SEM, both in decimal and hh:mm format, where appropriate.

	13-17 years/old	18-30 years/old	31-40 years/old	41-50 years/old	51-64 years/old	65-80 years/old
BMI	21.04 ± 0.69	22.92 ± 0.12	24.18 ± 0.07	24.85 ± 0.08	25.77 ± 0.09	27.10 ± 0.13
Averaged Time in Bed (h)	7.95 ± 0.04	7.84 ± 0.02	7.69 ± 0.02	7.58 ± 0.02	7.31 ± 0.03	7.53 ± 0.15
MBFbc (h)	4.88 ± 0.04	5.22 ± 0.02	4.46 ± 0.02	4.02 ± 0.02	3.94 ± 0.03	3.76 ± 0.14
(hh:mm)	04:52 ± 0:02	05:13 ± 0:01	04:27 ± 0:01	04:01 ± 0:01	03:56 ± 0:01	03:45 ± 0:08
Difference Mid Bed Time (FD-WD), social jet lag (h)	2.14 ± 0.04	1.84 ± 0.02	1.42 ± 0.02	1.21 ± 0.02	1.08 ± 0.03	0.54 ± 0.07
% with SJL (Difference Mid Bed Time (FD-WD)) > 2 hours	50.41	36.44	20.10	13.11	9.27	1.67
Commute time (min)	20.11 ± 0.59	30.68 ± 0.41	27.29 ± 0.39	28.33 ± 0.43	25.81 ± 0.60	19.77 ± 2.42
Time to get ready (min)	43.27 ± 0.74	49.61 ± 0.60	52.64 ± 0.67	57.15 ± 0.76	59.71 ± 1.10	59.52 ± 4.81
Work Start (h)	8.41 ± 0.03	9.65 ± 0.04	9.00 ± 0.04	8.72 ± 0.03	8.76 ± 0.05	9.28 ± 0.20
(hh:mm)	08:24 ± 0:01	09:39 ± 0:02	09:00 ± 0:02	08:43 ± 0:01	08:45 ± 0:03	09:16 ± 0:12
Work End (hh:mm)	14.93 ± 0.06	16.89 ± 0.05	17.20 ± 0.05	16.76 ± 0.05	16.77 ± 0.09	16.80 ± 0.44
(hh:mm)	14:55 ± 0:03	16:53 ± 0:03	17:12 ± 0:03	16:45 ± 0:03	16:46 ± 0:05	16:48 ± 0:26
Work-Natural Darkness Desynchronization (h)	0.01 ± 0.00	0.13 ± 0.01	0.13 ± 0.01	0.08 ± 0.01	0.11 ± 0.01	0.09 ± 0.04
Bed-Natural Darkness Desynchronization (h)	2.93 ± 0.04	3.46 ± 0.02	2.72 ± 0.02	2.30 ± 0.02	2.26 ± 0.04	2.22 ± 0.15
Bed-Work Desynchronization (h)	1.26 ± 0.03	0.90 ± 0.01	0.73 ± 0.01	0.62 ± 0.01	0.61 ± 0.02	0.29 ± 0.06
Three Times Score (A.U.)	0.12 ± 0.00	0.12 ± 0.00	0.10 ± 0.00	0.08 ± 0.00	0.08 ± 0.00	0.07 ± 0.01

BMI: Body Mass Index; MBFbc: modified version of the sleep corrected midsleep in free days (MSFsc); FD: free day; WD: work day.
